# ICTV Virus Taxonomy Profile: Fimoviridae 2024

**DOI:** 10.1099/jgv.0.001943

**Published:** 2024-05-02

**Authors:** Michele Digiaro, Toufic Elbeaino, Kenji Kubota, Francisco M. Ochoa-Coron, Susanne von Bargen

**Affiliations:** 1 CIHEAM-Istituto Agronomico Mediterraneo of Bari, Via Ceglie 9, 70010 Valenzano, Bari, Italy; 2 Institute for Plant Protection, NARO, 2-1-18 Kannondai, Tsukuba, Ibaraki 305-8666, Japan; 3 Oklahoma State University, Institute for Biosecurity and Microbial Forensics, 127 NRC, Stillwater, OK 74078, USA; 4 Humboldt-Universität zu Berlin Thaer-Institute of Agricultural and Horticultural Sciences, Lentzeallee 55/57, 14195, Berlin, Germany

**Keywords:** *Emaravirus*, *Fimoviridae*, ICTV Report, taxonomy

## Abstract

Members of the family *Fimoviridae *are plant viruses with a multipartite negative-sense enveloped RNA genome (−ssRNA), composed of 4–10 segments comprising 12.3–18.5 kb in total, within quasi-spherical virions. Fimoviruses are transmitted to plants by eriophyid mites and induce characteristic cytopathologies in their host plants, including double membrane-bound bodies in the cytoplasm of virus-infected cells. Most fimoviruses infect dicotyledonous plants, and many cause serious disease epidemics. This is a summary of the ICTV Report on the family *Fimoviridae*, which is available at ictv.global/report/fimoviridae.

## Virion

**Fig. 1.**
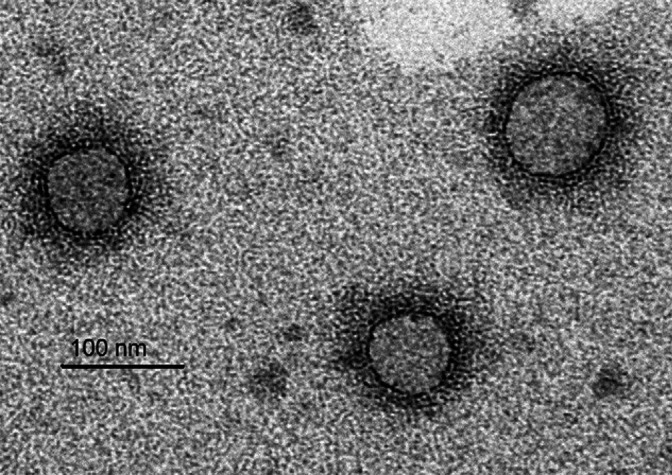
Immunosorbent electron micrograph of virions of European mountain ash ringspot-associated virus. Courtesy of Inga Ludenberg, University of Hamburg, Germany.

Fimoviruses have quasi-spherical, enveloped virions, with a diameter of 80–150 nm (Table 1, Fig. 1).

**Table 1.** Characteristics of members of the family *Fimoviridae*

**Example**	**fig mosaic virus isolate GR10 (RNA1: AM941711; RNA2: FM864225; RNA3: FM991954; RNA4: FM992851; RNA5: HE803826; RNA6: HE803827),species** ***Emaravirus fici*** **, genus** ***Emaravirus***
Virion	Quasi-spherical and enveloped with a diameter of 80–150 nm
Genome	4–10 segments of negative-sense RNA (12.3–18.5 kb in total)
Replication	Probably cytoplasmic
Translation	From capped mRNAs (produced by ‘cap snatching’ from host mRNAs), which are complementary to the vRNAs
Host range	Mainly dicotyledonous species (camellia, *Cercis*, fig, grapevine, kiwi, lilac, oak, pear, pigeonpea, pistachio, raspberry, rose, rowan, etc.) and a few monocotyledonous species (wheat, maize, ti). Some species are mechanically transmissible to herbaceous indicators
Taxonomy	Realm *Riboviria*, kingdom *Orthornavirae*, phylum *Negarnaviricota*, class *Bunyaviricetes*, order *Elliovirales*; the family includes the genus *Emaravirus* and >30 species

## Genome

**Fig. 2.**
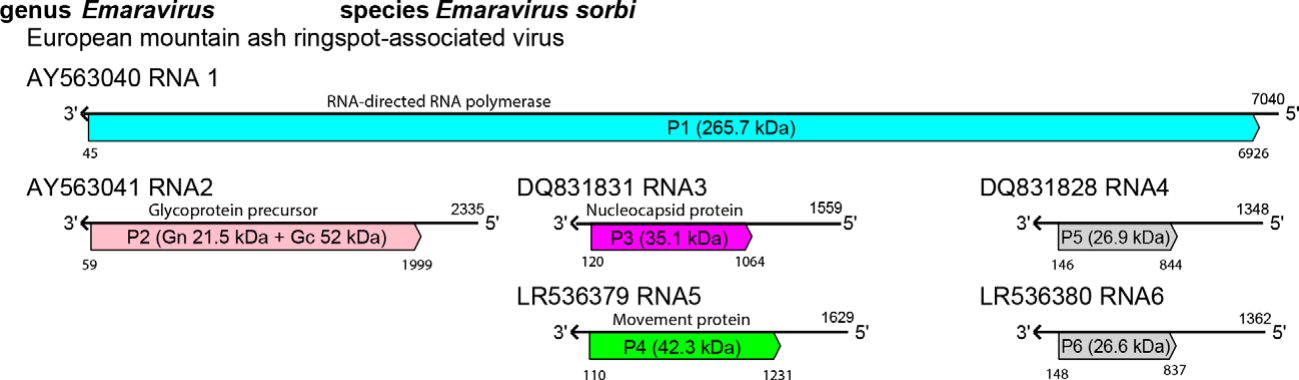
Genome organization of European mountain ash ringspot-associated virus. ORFs (coloured boxes) are annotated with protein function and estimated molecular mass. Grey colour; ORFs encoding proteins of unknown function.

The viral genome comprises 4 (palo verde broom virus, Camellia japonica-associated virus 2 and Ailanthus crinkle leaf-associated virus) to 10 (Perilla mosaic virus) segments of negative-sense RNA, comprising 12.3–18.5 kb in total [1,2]. The complementary strand of each genome RNA segment encodes a single protein (Fig. 2). From RNA1 to RNA4, the order of the encoded proteins is: the RNA-directed RNA polymerase (P1, 265–275.2 kDa); a glycoprotein precursor (P2, 68.2–82.9.6 kDa), which is predicted to cleave into two or three products; the nucleocapsid protein (P3, 29.5–36.0 kDa); and a putative movement protein (P4, about 35.4–43.6 kDa). The functions of the proteins encoded by RNAs 5–8 remain unknown. A possible role as a suppressor of RNA silencing of proteins encoded by RNA7 and RNA8 is hypothesized for High Plains wheat mosaic virus [3]. Some fimoviruses contain duplicated genome segments whose significance remains to be clarified. Genomic RNAs are not capped or polyadenylated and all contain almost perfectly complementary sequences (18–20 nt, depending on the RNA segment) at their 5′- and 3′-termini [4].

## Replication

Like other multipartite −ssRNA viruses, some fimoviruses use ‘cap snatching’ from host mRNAs to initiate transcription and facilitate translation of virus mRNAs [5,6]. Cap snatching involves binding of capped host mRNAs to the ribonucleoproteins, cleavage of these RNAs close to the 5′-cap by a viral endonuclease activity, and use of the short, capped fragments as primers for viral mRNA transcription. As for other bunyaviruses, the replication of fimoviruses probably occurs in the cytoplasm.

## Taxonomy

Current taxonomy: ictv.global/taxonomy. Viruses of the family *Fimoviridae* are distantly related to those of the families *Tospoviridae* and *Peribunyaviridae* in that they share: (i) a multipartite −ssRNA genome of 4–10 segments; (ii) high sequence identity with orthologous proteins of members of closely related virus families at equivalent genome positions in the first three RNAs (corresponding to L, M and S RNA segments), i.e. RNA-directed RNA polymerase (RNA1), putative glycoprotein precursor (RNA2) and putative nucleocapsid protein (RNA3); (iii) seven conserved motifs (preA–A–F) in the amino acid sequence of their RNA-directed RNA polymerase; (iv) enveloped virions; (v) stretches of nucleotides at both 5′- and 3′-termini of all RNA segments that are almost perfectly complementary to each other. These terminal sequences have several nucleotides conserved in all genomic RNAs of fimoviruses and are similar, but not identical, to those of members of the families *Hantaviridae* and *Peribunyaviridae*. Phylogenetic trees constructed with fimovirus RNA-directed RNA polymerase, glycoprotein precursor and nucleocapsid protein sequences display four main clusters [7].

## Resources

Full ICTV Report on the family *Fimoviridae*: ictv.global/report/fimoviridae.
