# Erratum ICTV virus taxonomy profile: Fimoviridae 2024

**DOI:** 10.1099/jgv.0.001992

**Published:** 2024-05-17

**Authors:** Michele Digiaro, Toufic Elbeaino, Kenji Kubota, Francisco M. Ochoa-Corona, Susanne von Bargen

**Affiliations:** 1 CIHEAM-Istituto Agronomico Mediterraneo of Bari, Via Ceglie 9, 70010 Valenzano, Bari, Italy; 2 Institute for Plant Protection, NARO, 2-1-18 Kannondai, Tsukuba, Ibaraki 305-8666, Japan; 3 Oklahoma State University, Institute for Biosecurity and Microbial Forensics, 127 NRC, Stillwater, OK 74078, USA; 4 Humboldt-Universität zu Berlin Thaer-Institute of Agricultural and Horticultural Sciences, Lentzeallee 55/57, 14195, Berlin, Germany

In the published version of this article, one of the authors' names has been misspelled as 'Francisco M. Ochoa-Coron'. The correct name of the author is Francisco M. Ochoa-Corona. The original version of this has also been updated to reflect this change.

The Microbiology Society apologizes for any inconvenience this oversight may have caused.

